# Effectiveness of nurse‐led counselling and education on self‐efficacy of patients with acute coronary syndrome: A randomized controlled trial

**DOI:** 10.1002/nop2.1129

**Published:** 2021-11-12

**Authors:** Hossein Bagheri, Sara Shakeri, Ali‐Mohammad Nazari, Shahrbanoo Goli, Mahboobeh Khajeh, Abbas Mardani, Zeljko Vlaisavljevic

**Affiliations:** ^1^ Department of Nursing School of Nursing and Midwifery Shahroud University of Medical Sciences Shahroud Iran; ^2^ Student Research Committee School of Nursing and Midwifery Shahroud University of Medical Sciences Shahroud Iran; ^3^ Department of Midwifery Faculty of Nursing and Midwifery Shahroud University of Medical Sciences Shahroud Iran; ^4^ Center for Health Related Social and Behavioral Sciences Research Shahroud University of Medical Sciences Shahroud Iran; ^5^ Nursing Care Research Center Department of Medical Surgical Nursing School of Nursing and Midwifery Iran University of Medical Sciences Tehran Iran; ^6^ University Clinical Center of Serbia Clinic for Gastroenterology and Hepatoligia Belgarade Serbia; ^7^ Medical School of Vocational Studies Medika, Department of Nursing Belgarade Serbia

**Keywords:** acute coronary syndrome, counselling, education, nurse, person‐centred care, self‐efficacy

## Abstract

**Aim:**

Adherence to lifestyle recommendations, medical regimens and cardiac rehabilitation is poor among patients with acute coronary syndrome. The aim of this study was to examine the effect of nurse‐led counselling and education using a person‐centred care approach on short‐term cardiac self‐efficacy in patients with acute coronary syndrome.

**Design:**

A parallel, two‐armed, randomized controlled trial was conducted.

**Methods:**

One hundred twenty patients who were hospitalized with diagnosis of acute coronary syndrome were selected and randomly assigned into intervention (*n* = 60) or control (*n* = 60) groups. In the intervention group, in addition to routine care, the nurse‐led counselling and education programme included two face‐to‐face sessions, two telephone counselling and education sessions, using the person‐centred care approach. Participants in the control group received only routine care. Data were collected using the cardiac self‐efficacy scale before the intervention and 1 month after discharge.

**Results:**

After the intervention, we found that cardiac self‐efficacy, including the perceived self‐efficacy to control symptoms and maintain function, was statistically significantly higher in the intervention group than the control group.

## 1 INTRODUCTION

Acute coronary syndrome (ACS) is a type of coronary heart disease. It is responsible for around one‐third of deaths of people over 35 (Sanchis‐Gomar et al., 2016). ACS—which encompasses three acute conditions, including ST‐elevation myocardial infarction (STEMI), non‐ST‐elevation myocardial infarction (NSTEMI) and unstable angina—is the result of endothelial dysfunction or unstable atheromatous plaques with transient or permanent thrombotic blockage of the coronary artery, leading to myocardial ischaemia and infarction (Atherton et al., 2018; Collet et al., 2020). Patients with ACS typically suffer from multiple problems including pain (Chen et al., 2018), changes in the tissue blood flow (Liang et al., 2021), intolerance to physical activity (Rymuza et al., 2019), ineffective adaptation to the disease, and severe anxiety and depressive symptoms (Serrano‐Rosa et al., 2021), which have a negative impact on patients’ quality of life. The ideal management of ACS following the postacute period involves controlling risk factors such as diabetes, hypertension, atherogenic lipid profile, obesity and lifestyle modifications including promoting physical activity, dietary changes and smoking cessation (Bhagwat et al., 2016; Candelaria et al., 2020; Landmesser et al., 2020).

Lifestyle modifications and medical regimes help patients with ACS reduce cardiac risk factors and prevent further heart attacks (Steca et al., 2017). However, adherence to lifestyle recommendations, medical regimens and cardiac rehabilitation is poor (Fors et al., 2016). One of the key elements to empower patients is to improve their self‐efficacy (Boroumand & Moeini, 2016). The notion of self‐efficacy concerns patients’ belief in their capability to modify the incidents that affect their lives (Bandura, 1977). Self‐efficacy could have statistically significant implications for recovery because patients who feel confident and capable modify their behaviour are more probably to reach their treatment goals (Vaughan‐Johnston & Jacobson, 2020). There is strong evidence that supports a positive relationship between self‐efficacy and the health of patients with ACS (Barham et al., 2019). In addition, higher self‐efficacy is associated with better quality of life in patients with heart failure (Baradaranfard et al., 2018).

Counselling is a learning process that takes place through a relationship between two individuals (Geldard et al., 2017). The counsellor has scientific skills and competencies to meet patients’ needs. A counsellor can help a patient to learn more about themselves and determine what goals are realistic (Thompson, 2015). Counselling can help patients to make the best decision on how to act appropriately in difficult situations (Karimlou et al., 2019). The use of counselling and education programmes by healthcare providers, including nurses, can improve the health and the quality of life of patients (Wang et al., 2016). During counselling, the patient receives the information that helps them to understand how to best fulfil their needs (Wang et al., 2016). In addition, with follow‐up of the patients and discussing how they are managing self‐care at home, nurses ensure that understand all the issues that patients encounter. This, in turn, allows the nurses to provide the necessary guidelines which help patients resolve the aforementioned issues (Townsend & Morgan, 2017).

Previous studies have shown that patients’ counselling and education, coupled with person‐centred care (PCC) approach, can improve self‐efficacy, facilitate patients' rehabilitation and enhance their self‐management. This seems to be especially true in cases when patients struggle with heart diseases (Ekman et al., 2012; Fors et al., 2016). The World Health Organization has identified PCC as a core component of high‐quality care for patients with chronic diseases (Santana et al., 2018). A central part of PCC is that the healthcare provider and patient jointly develop a care plan by identifying resources and potential barriers for each patient (Byrne et al., 2020).

Previous studies documented contradictory findings of the effectiveness of nurse‐led counselling and education on the self‐efficacy of ACS patients. For instance, Weibel et al.’s (2016) study showed pre‐discharge education and counselling sessions may improve the self‐efficacy of patients with ACS. However, the study used a small sample size (40 participants) and cardiac self‐efficacy was measured at short intervals including before intervention and immediately after the intervention. The short intervals mean that the study did not measure the full effect of the intervention or focus on its lasting effects (Weibel et al., 2016). Another study used a quasi‐experimental pre‐test–post‐test design and had a sample size of 62 patients. It concluded pre‐discharge education, counselling and two telephone‐based follow‐up counselling sessions have no effect on the self‐efficacy of ACS patients in the long term (Shim & Hwang, 2017). However, the participants were only limited to middle‐aged patients and the study failed to specify what routine care received by the patients in the control group (Shim & Hwang, 2017).

Furthermore, there are many education strategies that use for various clinical outcomes in ACS patients. An umbrella review that evaluated health education for patients with ACS and type two diabetes mellitus concluded that different health education strategies including face‐to‐face sessions and telephone or via web education can be effective in reducing smoking and admissions for ACS patients (Liu et al., 2017). However, a recent systematic review investigated the clinical usefulness of hospital discharge education strategies in patients with ACS and three randomized controlled trials included in the review. Interventions contained educational sessions that targeted participants' needs and were providing risk factors and lifestyle modification and cardioprotective risk factor advice and inviting them to attend a cardiac rehabilitation programme. The review found little to no evidence for the effectiveness of current discharge practices on in relation to medication adherence, attendance to the cardiac rehabilitation programme and physical activity rate (Kourbelis et al., 2020). Given that high self‐efficacy can improve clinical outcomes in patients with ACS and there are contradictory findings in the literature (Barham et al., 2019), more research with larger sample size and rigorous methodology is necessary.

With the reduction in the length of hospital stay for patients with ACS, early intervention becomes pivotal. Nurses can use education to increase their patients' knowledge about the disease and treatments. In addition, they can use counselling to help patients make the best decision about the appropriate actions they should follow and help them understand how to cope more effectively. The aim of the study was to investigate the effect of nurse‐led counselling and education using the PCC approach on the short‐term cardiac self‐efficacy of patients with ACS.

## 2 METHODS

### 2.1 Research questions and hypotheses

The research question for this study was as follows: Does nurse‐led counselling and education using the PCC approach improve the short‐term cardiac self‐efficacy of patients with ACS?

The research hypotheses for the current study were as follows:

- H_0_: A nurse‐led counselling and education programme using the PCC approach has no effect on the short‐term cardiac self‐efficacy in patients with ACS.
- H_A_: A nurse‐led counselling and education programme using the PCC approach has effect on the short‐term cardiac self‐efficacy in patients with ACS.

Furthermore, the hypotheses for demographic variables were as follows:

- H_0_: Demographic variables are homogeneous between the intervention and the control group.
- H_A_: Demographic variables are not homogeneous between the intervention and the control group.

### 2.2 Design

This study used a parallel, two‐armed, randomized controlled trial design. Patients with ACS who were admitted to a cardiac department of a referral teaching hospital in Shahroud, Iran from October 2018–July 2019 participated in the study.

### 2.3 Participants and sampling

The eligibility criteria for involvement in this study were as follows: (a) aged <75 years, (b) hospitalized with diagnosis of ACS, (c) received a score of less than the mean (<26) from the cardiac self‐efficacy scale which indicates poor cardiac self‐efficacy (Sullivan et al., 1998) and (d) the ability to speak and understand Farsi. The exclusion criteria in the study included as follows: (a) a history of cognitive or cognitive and psychological disorders based on the patient's medical file and self‐report, (b) being on a planned heart surgery list such as coronary artery bypass grafting, (c) alcohol and/or drug addiction and (d) discontinued the intervention or follow‐up.

The required sample size according to Varaei et al.’s (2017) for with 5% type one error and 80% power estimated at 60 participants in each group for this study. Sampling was done through a convenience sampling method. Firstly, the patients admitted to a cardiac department at the study hospital with ACS were reviewed to find eligible patients. To ensure that appropriate patients were enrolled, the International Classification of Diseases (ICD) codes in their medical records were checked.

**FIGURE 1:**
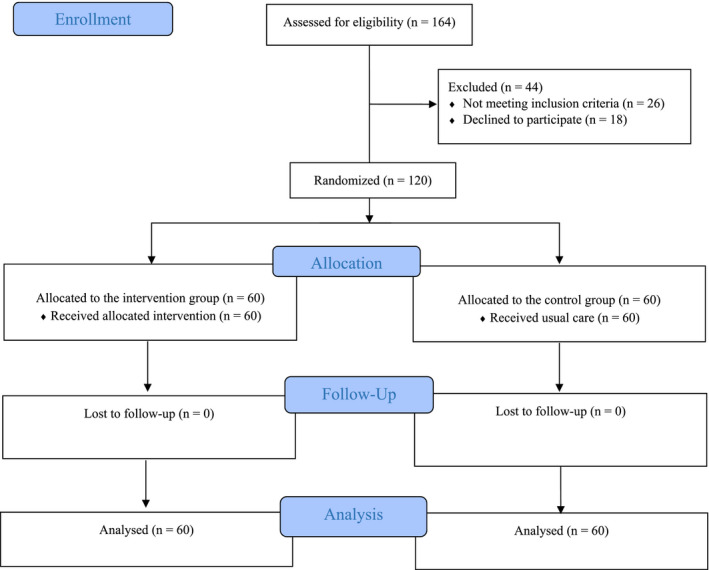
Process of the study according to the CONSORT flow diagram (2010)

Secondly, the eligible participants were randomly assigned to the study groups using the block randomization design as follows: The randomization design used a random allocation sequence with a block size of four (30 blocks) for two groups (A and B) determined by the study statistical advisor (SG) using SPSS syntax. Next, the eligible patients were allocated to the intervention and the control groups based on the determined sequence by the second author (SS). The concealment procedure was performed using sequentially numbered sealed opaque envelopes, in which cards with letters A “representing the intervention group” or B “representing the control group” indicating the allocation sequence were placed (Figure 1). In this study, the participants and the data collector (SS) were not blinded and only the data analyser (SG) were blind to the group assignments.

### 2.4 Variables and data collection tools

In the present study, the independent variable was counselling and education programme and dependent variable were cardiac self‐efficacy. Statistically significant extraneous variables affecting cardiac self‐efficacy including older age, amount of cardiac self‐efficacy, cognitive and psychological disorders, and addiction were identified through a review of the literature (Fors et al., 2016; Shim & Hwang, 2017; Weibel et al., 2016). The possible influence of these variables on cardiac self‐efficacy was minimized through determine accurate inclusion and exclusion criteria. In addition, demographic variables for evaluating the homogeneity in two groups of the study were collected.

#### 2.4.1 Demographic data questionnaire

A demographic data questionnaire was designed by the researchers through a review of the literature to gather demographic characteristics of the participants. This questionnaire included questions about a participant's age, gender, body mass index (BMI), marital status, education, occupation, economic status and health insurance status. Content and face validity methods were used to confirm the validity of this questionnaire. The questionnaire was provided to 10 patients with ACS (for evaluating face validity) and 10 nursing faculty members (for evaluating content validity). After gathering their suggestions, corrective feedback was applied. This questionnaire was completed at the beginning of the study in the cardiac department by the participants or by the researcher (SS) through interviewing the participants who were unable to read.

#### 2.4.2 Cardiac self‐efficacy scale

Cardiac self‐efficacy scale was developed to measure self‐efficacy related to heart disease (Sullivan et al., 1998). This scale has 13 questions in two main dimensions including control symptoms (eight questions) and maintain function (five questions). It evaluates the level of self‐efficacy in patients using a 5‐point Likert scale ranging from 0 (i.e. not at all confident) to 4 (i.e. strongly confident). The score range for the control symptoms dimension is from 0–32 and for the maintain function dimension is from 0–20. Accordingly, the total self‐efficacy score ranges from 0–52, and higher scores represented better‐perceived self‐efficacy. In addition, a score of less than the mean (<26) indicated poor cardiac self‐efficacy. In the study by Sullivan et al. (1998), Cronbach’s alpha coefficient for the original English version of this scale was above 0.87–0.90. The validity of the Farsi version of this scale was confirmed using content validity method (content validity index = 91.33%), and reliability was confirmed with Cronbach's alpha coefficient 0.97 (Varaei et al., 2017). In the current study, Cronbach's alpha coefficient was calculated 0.88.

The participants, or the researcher (SS) by interviewing the participants who were unable to read, completed this questionnaire before intervention at the cardiac department and 1 month after discharge from the hospital at their place of residence.

### 2.5 Intervention

The participants in the control group received routine care. Routine care encompassed information and education provided by the nursing staff during discharge through verbal education and an information pamphlet about diet, drugs and cardiac symptoms that require special attention. In addition, prior to discharge, the nursing staff highlighted individual risk factors for each patient and gave them appropriate recommendations.

Nurse‐led counselling and education were performed using the PCC approach for participants in the intervention group in addition to the routine care by the second researcher (SS). It was a programme based on cardiac self‐efficacy improvement strategies. The intervention programme was developed by the research team—consisting of experts in the field of heart disease. The research team also relied on the steps proposed by van Meijel et al. (2004) that included problem description, accumulation of building blocks for intervention design, design of intervention and intervention validation. The validity of the intervention programme was confirmed through face and content validity methods. The programme was provided to 10 experts in the field of heart disease care to determine face and content validity. After gathering the recommendations, corrective feedback was applied.

The counselling and education process was carried out in three phases as follows:

Phase Ⅰ: The main purpose of this phase was to obtain information related to the patient's everyday life before hospitalization, symptoms, resources, barriers and their goals/motivation. This stage was performed in the cardiac ward in a specially reserved room one day before discharge and took about 20–30 min. Additionally, the patient was educated through a booklet and also face‐to‐face conversations which focused on the risk factors and symptoms of heart diseases, effects of the disease on work and life and ways to overcome them, as well as sexual relationships and social activities, diet, drugs and complications of non‐compliance with treatment.
Phase Ⅱ: In the second step, conducted on the day of discharge, counselling recommendations, based on identified patient's needs, preferences, resources and barriers, were incorporated into the care programme. The recommendations were provided in written form and comprised measures required in‐home care and rehabilitation programmes. In addition, the patients were told how to modify adjustable risk factors.
Phase Ⅲ: The next step was telephone counselling, education and follow‐up sessions. These were conducted one and three weeks after discharge. In this phase, which lasted 10 min for each contact, the patient's questions and concerns were answered and they were asked about the treatment follow‐up, the use of medications, and observation of the diet and self‐care. Thus, necessary counselling recommendations and education were made according to the needs of patients. In addition, patients were encouraged to adhere to the care programme and recommendations.

### 2.6 Ethical considerations

The Medical Ethics Committee of Shahroud University of Medical Sciences provided approval for the research proposal (decree code: IR.SHMU.REC.1396.202). The protocol of the study was registered on the Iranian Registry of Clinical Trials (IRCT) website under the code of IRCT20100114003064N9. All potential participants received information about the aims of this study and methods. In addition, they could leave the study at any time without being penalized or influencing the quality of their care. Finally, the confidentiality of collected data was guaranteed. Participants wishing to participate in the study signed the consent form which contained all the necessary information about the study.

### 2.7 Data analysis

The SPSS version 25 was used for data analysis. Intervention and control group data were summarized descriptively using frequency and per cent for categorical variables and mean and standard deviation (*SD*) for continuous variables. The homogeneity of the study groups was compared applying independent sample *t* test for age and BMI; chi‐square test for gender, marital status, occupation, educational level and economic status; and Fisher's exact test for health insurance. The independent sample *t* test and paired sample *t* test were used to compare cardiac self‐efficacy scores between and in the study groups, respectively. An alpha level of 0.05 was applied for inferential statistical tests.

## 3 RESULTS

Out of 164 patients with ACS who were reviewed in terms of eligibility criteria, 120 patients were randomly assigned to intervention (*n* = 60) and control (*n* = 60) groups. None of the participants dropped out during the follow‐up. Therefore, gathered data from all 60 participants in each of the groups were applied for analysis (Figure 1).

### 3.1 Demographic characteristics

The mean age of the participants was 57.03 and 59.28 years in the intervention and control groups, respectively. In the intervention group, 52.2% of participants and in the control group 60% were female. The participants' mean BMI in the intervention and control groups was 24.56 and 25.11, respectively. The majority of participants in both groups were married (86.7% in both groups). Other demographic information is given in Table 1. According the results of inferential statistical tests that are presented in Table 1, there were no statistically significant differences in terms of demographic variables in the groups (*p* > .05). Therefore, the null hypothesis was accepted which indicates that two study groups were homogeneous in view of demographic variables.

**TABLE 1:** Demographic characteristics of the patients in the groups

Variable	Study groups		*p* Value
	Intervention group *n* (%)	Control group *n* (%)	
Age; year, mean (*SD*)	57.03 (8.01)	59.28 (9.27)	.15*
Gender			
Male	28 (46.7)	24 (40)	.46**
Female	32 (52.2)	36 (60)	
BMI, kg, mean (*SD*)	24.56 (0.89)	25.11 (1)	.50*
Marital status			
Married	52 (86.7)	52 (86.7)	
Single	8 (13.3)	8 (13.3)	1**
Occupation			
Employed	28 (46.7)	22 (36.7)	
Unemployed	32 (53.3)	38 (63.3)	.28**
Education level			
Unable to read	21 (35)	27 (45)	
Elementary and higher	39 (65)	33 (55)	.24**
Economic status (self‐report)			
Sufficient	7 (11.7)	2 (3.3)	.12**
Relatively sufficient	31 (51.7)	39 (65)	
Not sufficient	22 (36.6)	19 (31.7)	
Health insurance			
Yes	58 (96.7)	59 (98.7)	.99***
No	2 (3.3)	1 (1.3)	

* Independent sample *t* test; ^**^Chi‐square test; ^***^Fisher exact test.

### 3.2 Cardiac self‐efficacy

As shown in Table 2, before the intervention, there was no statistically significant difference between the two study groups in the mean scores of control symptoms dimension (*p* = .94), maintain function dimension (*p* = .52) and total cardiac self‐efficacy (*p* = .73).

**TABLE 2:** Self‐efficacy in the patients before intervention and one month after discharge in the study groups

Variable	Time	Intervention group		Control group		Between group comparison *p*‐value^*^
		Mean	*SD*	Mean	*SD*	
Control symptoms	Before intervention	10.66	3.54	10.61	3.71	.94
	one month after discharge	19.40	3.19	13.73	4.45	<.001
Intragroup comparison	*p*‐value^**^	<.001		<.001		
Maintain functioning	Before intervention	9.85	1.84	9.61	2.12	.52
	One month after discharge	12.83	2.40	10.20	2.88	<.001
Intragroup comparison	*p*‐value^**^	<.001		.08		
Total self‐efficacy	Before intervention	20.51	4.21	20.23	4.85	.73
	One month after discharge	32.23	4.91	23.93	6.66	<.001
Intragroup comparison	*p*‐value^**^	<.001		<.001		

^*^Independent samples *t* test; ^**^Paired samples *t* test.

In the intragroup comparisons of cardiac self‐efficacy before and 1 month after discharge, in the intervention group, the mean scores of control symptom dimension and maintain function dimension and total cardiac self‐efficacy significantly increased after the intervention programme (*p* < .001). Although, in the control group, 1 month after discharge control symptoms dimension and cardiac total self‐efficacy significantly improved compared with before the intervention (*p* < .001), the mean increase in the control group was lower than the intervention (Table 2).

Comparison of control symptoms dimension and maintain function dimension and total cardiac self‐efficacy between the groups 1 month after the discharge demonstrated that participants in the intervention group who received nurse‐led counselling and education programme using the PCC approach had statistically significant improvement than those in the control group (*p* < .001) (Table 2). Therefore, on the basis of the substantial evidence, the null hypothesis was rejected. Accordingly, it can be concluded that applying nurse‐led counselling and education programme using the PCC approach was effective for improving cardiac self‐efficacy.

**FIGURE 2:**
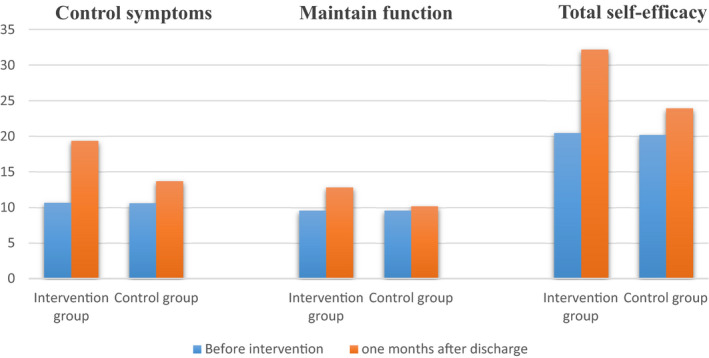
Comparison of the self‐efficacy of participants in two study groups

The trend of changes in participants' cardiac self‐efficacy before the intervention and 1 month after discharge was illustrated in Figure 2.

## 4 DISCUSSION

The effect of the nurse‐led counselling and education programme on the short‐term cardiac self‐efficacy of ACS patients was investigated in this research.

Increase in self‐efficacy is the foundation for future behavioural and lifestyle changes (Vaughan‐Johnston & Jacobson, 2020). Previous literature suggested that improved self‐efficacy has a statistically significant impact on the health behaviour of patients. For instance, patients with improved self‐efficacy were more probably to regularly exercise and engage in various programmes promoting physical activity. Additionally, such patients were also more probably to make better food choices (Castillo‐Mayén et al., 2020). The findings of the present study showed considerable improvements in short‐term perceived self‐efficacy to control symptoms and maintain function, and total cardiac self‐efficacy of participants in the intervention group compared with the control group. Consistent with the current study results, Weibel et al. (2016) in a pilot randomized controlled trial study found that delivering early education and counselling to patients with ACS led to the improvement of their short‐term perceived self‐efficacy to control symptoms and total self‐efficacy. However, in the mentioned study, perceived self‐efficacy to maintain function did not improve (Weibel et al., 2016), which is inconsistent with the findings of the present study. The reason for such inconsistency can be explained by the differences in the type of intervention. They conducted only education sessions in the pre‐discharge period, but in the current study, in addition to pre‐discharge counselling and education, two telephone counselling and follow‐up sessions were conducted during 1 month after the discharge.

According to the present study findings, nurse‐led counselling and education improved short‐term perceived self‐efficacy to control symptoms in ACS patients. It is well documented that patients with the optimal levels of knowledge of ACS symptoms have the capability to translate their knowledge to actions and behaviour changes in the presence of a health threat (O’Brien et al., 2014). The effect of nursing education and counselling has been explored on outcomes of other patients, which are closely related to cardiac self‐efficacy. For example, a quasi‐experimental study revealed that a nurse‐led education programme prior to discharge could improve the level of knowledge, attitudes and beliefs of patients in response to ACS symptoms 1 month after the education (Darsin Singh et al., 2018). In addition, McKinley et al. (2009) in a large‐scale randomized controlled trial found that a 40‐min individual, face‐to‐face educative counselling session could improve ACS patients’ knowledge, attitude and beliefs about symptoms 3 and 12 months after the intervention.

Some of the previous studies reached different conclusions. For instance, a quasi‐experimental study evidenced that provision of nurse‐led education and counselling prior to discharge, followed with two telephone follow‐ups sessions, had no effect on the long‐term self‐efficacy of ACS patients in 3 and 12 months after discharge (Shim & Hwang, 2017). However, the measurement time of self‐efficacy in this study was only limited to 1 month after discharge which could explain the difference in the results. This also may be due to the fact that patients with ACS gradually develop strategies to manage their symptoms and adjust to living with the disease (Nesterak & Bardashevska, 2019), which can reduce the differences between the intervention and control group participants in the studies over time. Nonetheless, the nurse‐led counselling and education appears to have accelerated adjustment, as showed by the present study’s 1‐month findings, which may have statistically significant implications for ACS patient recovery. For instance, self‐efficacy has been demonstrated to be related to several useful outcomes during cardiac recovery, including strengthen self‐management behaviours, encourage lifestyle modification, improve adherence to medication, enhance physical activity, and improve psychological well‐being and quality of life (Barham et al., 2019; Ha et al., 2018).

The intervention programme used in the current study represented the PCC approach (Byrne et al., 2020). The designed intervention programme did not only encompass cardiac medical knowledge and evidence‐based finding. Instead, but it also incorporated the individual resources, needs and barriers of the ACS patients into the intervention programme. Similar to the results of the present study, two studies revealed that applying person‐centred intervention improves self‐efficacy in patients with ACS after hospitalization (Fors et al., 2016; Weibel et al., 2016). In addition to the positive effect of PCC in patients with ACS, the efficacy of applying this approach has been documented in patients with chronic heart failure (Ekman et al., 2012; Hansson et al., 2016). Ekman et al.'s (2012) study found that PCC in patients with chronic heart failure caused a shortening length of hospital stay and maintaining function without increasing readmission risks or deteriorating health‐related quality of life.

### 4.1 Limitations

There are some limitations to this study, which ought to be considered when interpreting its results. Firstly, as the main limitation, the participants of the study were <75 and recruited from a single academic hospital; therefore, the results were not generalizable to all ACS patients in Iran and other countries. Secondly, the short‐term self‐efficacy (up to 1‐month postdischarge) was measured in this study, indicating that the results may be subject to change over a longer period. Thirdly, the after‐intervention evaluation of the participants' cardiac self‐efficacy was performed one week after the second telephone counselling and education session. The short time after the telephone counselling and education may mean that it is impossible to measure the full effect of the intervention. Fourthly, providing counselling and education to the patients and collecting data was performed by the same researcher, which could potentially be a source of bias. Fifthly, in this study, the data were gathered using a self‐report scale, and physiological parameters or clinical outcomes were not considered. Thus, we recommend that future studies include more parameters or outcomes, such as emergency department visits, readmission, and mortality over a longer period. Lastly, the personality characteristics of the researcher who provided counselling and education to patients might stimulate or prevent patients to learn, but this confounding variable was not evaluated.

### 4.2 Implications and recommendations for practice

According to the findings of the current study, the nurse‐led counselling and education programme using PCC is an effective strategy to improve the short‐term cardiac self‐efficacy of patients with ACS. Therefore, the intervention programme will improve and accelerate cardiac self‐efficacy if incorporated into the care plan of ACS patients. However, this change in the care plan of ACS patients would need a higher nurse‐patient ratio and special training for cardiac department nurses. Nevertheless, we are aware that huge nursing and resource shortages, which are particularly high in developing countries such as Iran (Nasirizad Moghadam et al., 2021; Shamsi & Peyravi, 2020), could prevent applying such nurse‐led counselling and education programmes. Therefore, it is recommended that healthcare organizations use a critical approach and analyse the costs and benefits when deciding on the necessary arrangements for the implementation of such programmes to improve the quality of patient care.

## 5 CONCLUSION

Nurses are well placed to provide care to patients with cardiac issues and require evidence‐based findings to improve their practice. The findings of the present study suggest that the provision of a nurse‐led counselling and education programme using the PCC approach can improve the short‐term cardiac self‐efficacy of patients with ACS. Further studies are needed not just to replicate our findings, but also to provide longitudinal data about the effect of nurse‐led counselling on long‐term cardiac self‐efficacy, objective clinical outcomes and preventing secondary adverse events in patients with ACS.

## CONFLICT OF INTEREST

No conflict of interest is declared by the authors.

## AUTHORS' CONTRIBUTIONS

HB, SS, AMN and MK involved in conceptualization. HB, SS and MK involved in methodology. SG and AM involved in formal analysis and investigation. HB, SS, AM and ZV involved in writing—original draft preparation. HB, AM and ZV writing—review and editing. HB involved in funding acquisition and supervision. HB and SS involved in resources. All authors reviewed the manuscript.

## ETHICS APPROVAL

The protocol of this study was approved by the Ethics Committee of the Shahroud University of Medical Sciences under the code of IR.SHMU.REC.1396.202.

## Data Availability

The data sets used for the current study are available from the corresponding author on reasonable request.
